# WhatsApp Linking Lilongwe, Malawi to Los Angeles: Impacting Medical Education and Clinical Management

**DOI:** 10.5334/aogh.3156

**Published:** 2021-02-18

**Authors:** Marguerite Thorp, Kara-Lee Pool, Christopher Tymchuk, Faysal Saab

**Affiliations:** 1 Department of Medicine, University of California, Los Angeles, US; 2 RAD-AID International, US; 3 Department of Medicine – Division of Infectious Diseases, University of California, Los Angeles, US

## Abstract

**Background:**

Subspecialty expertise is often lacking in clinical environments in low-resource settings. As a result, medically complicated patients can receive suboptimal care, local clinicians can feel inadequately supported, and global health engagements can be difficult for medical trainees accustomed to more expert supervision at their home institutions.

**Objective:**

We created WhatsApp Messenger discussion groups to connect subspecialists at the University of California, Los Angeles (UCLA) David Geffen School of Medicine with clinicians and rotating global health residents at Partners in Hope (PIH) Medical Center in Lilongwe, Malawi.

**Methods:**

Case submitters and subspecialist respondents were surveyed about their experience in the discussion groups.

**Findings:**

Over a three-year period, 95 cases were discussed in ten subspecialty groups, with dermatology and radiology/pulmonology receiving the most submissions. Participants were surveyed and reported excellent educational outcomes; large majorities of both case submitters (89%) and experts (71%) agreed or strongly agreed that the case discussions improved their medical education. The surveys also suggested positive impact on medical management decisions and patient outcomes. The major challenge to our intervention was low utilization of this resource by Malawian clinicians in comparison to medical residents. We hope to further address the barriers to participation and adapt the intervention to better support our Malawian colleagues.

**Conclusion:**

Because the discussion groups are free to create and require very little maintenance, this intervention can be easily replicated at other institutions looking to augment their global health educational engagements and support their clinical partners abroad.

## Introduction

Health care in low-resource settings is often provided by clinicians with strong practical skills but limited subspecialty training. In Malawi, health centers and hospitals are staffed primarily by clinical officers, who complete four years of post-secondary training in medicine and surgery, including a mandatory one-year internship. Where resources are available, they may be supervised by medical officers, who complete six years in a professional degree program (Bachelor of Medicine/Bachelor of Surgery, or MBBS), which also includes one year of internship. Post-graduate residency programs for MBBS candidates are few and often financially prohibitive.

While many patients can be cared for by providers with basic training in common conditions such as HIV, tuberculosis, and malaria, many others require expert care. Limited access to subspecialty expertise is compounded by sparse diagnostic and treatment modalities. When faced with challenging cases, clinicians in these environments may feel ill-prepared and frustrated by this expertise gap. This can increase burnout, decrease clinician efficacy, and even result in leaving the healthcare workforce [1,2]. The expertise gap is even more jarring when encountered by medical trainees from developed countries; those who participate in global health rotations can be unsettled by the lack of expert supervision, especially in contrast to the well-resourced teaching hospitals where they train.

We sought to close this expertise gap with the use of mobile instant messaging (MIM) groups for Malawian clinicians and American medical residents rotating at a medical center in Malawi. MIM technology has been widely adopted for personal and professional use in Malawi, including in the health sector [3,4,5]. Many projects in other settings have connected physicians in order to overcome gaps in resources and expertise. Some are clearly intended to improve clinical care by providing consultant telehealth services [6,7,8,9,10,11,12]. Others create online learning modules for trainees or educate clinicians on advanced topics and rare cases [13,14,15,16]. Our intervention hybridized these goals — we hoped that detailed case discussions would provide an educational opportunity for clinicians working in Malawi, allowing them to make more appropriate management decisions that could result in improved patient care and outcomes.

By leveraging newer technology, we were able to avoid many challenges of previous networks such as poor image quality and slow upload speeds [17,18]. Most networks utilize “store and forward” technology, with information and images uploaded first and reviewed at a later time [19]. The distinction between store-and-forward and live connection has become less significant with the advent of MIM [20,21]. Given the 10-hour time difference, responses in our groups sometimes took up to 24 hours, but if requests arrived during working hours in Los Angeles, replies were often immediate. This allowed for real-time dialogue between requesters and experts, which was very limited prior to MIM.

Other authors have identified the need for protection of patient privacy in telehealth [20,22], but previous interventions have had inconsistent records with execution. Our affiliation with a United States academic institution made these concerns even more salient. We implemented strong protections including de-identifying cases, coding for HIV status in accordance with Malawian guidelines (using “R/NR” for “reactive/non-reactive”), obtaining verbal consent from patients for all communications, and escalating to written consent if any case discussions required identifiable information such as photos of the face. WhatsApp’s uniquely encrypted platform provided additional security.

## Program Description

### Setting

Partners in Hope (PIH) is a non-governmental medical center in Lilongwe, Malawi with whom the University of California, Los Angeles (UCLA) has had a long-standing research and education partnership. PIH cares for over 10,000 patients with HIV and other medical conditions in a free clinic, a paid clinic that helps offset the costs of the free clinic, and a 19-bed inpatient ward. The clinics and ward are staffed by four Malawian clinical officers and six medical officers, operating under the supervision of two senior physicians. Approximately eight times annually, small groups of internal medicine residents and a supervising faculty member from UCLA rotate at PIH for three-week blocks. This academic relationship has been further strengthened by long-term UCLA faculty presence over the past twelve years and visits to UCLA by our Malawian colleagues. The result has been a mutually fruitful collaboration, fostering opportunities for innovation in medical education, research, and clinical training.

### Intervention

To connect providers at PIH with subspecialists at UCLA, we created ten unique instant messaging groups, one for each specialty, via the mobile application WhatsApp (***Table 1***). Radiology and pulmonology were combined because the interpretation of chest radiographs was a common topic. Subspecialist attendings and fellows, as well as senior residents in non-internal medicine specialties such as neurology, dermatology, and radiology, were added to each group as experts. Before joining the groups, experts received communication guidelines, standard expectations regarding response times, and lists of medications and diagnostic modalities typically available in Malawi.

**Table 1:** Cases submitted by subspecialty topic.


SUBSPECIALTY GROUP	CASES WITH COMPLETED SURVEYS	CASES WITHOUT COMPLETED SURVEYS	CASES SUBMITTED BY STUDY TEAM (SURVEY EXCLUDED)

Cardiology	7		9

Dermatology	16		4

Endocrinology	4		

Gastroenterology	0	2	

Hematology & Oncology	1		

Infectious Disease	8		

Nephrology	0	1	

Neurology	7		3

Radiology & Pulmonology	22		7

Rheumatology	4		

Total	69	3	23

Total cases	95		


PIH clinicians and visiting UCLA residents (requesters) initiated discussion of clinical cases as needed, requesting input from the appropriate WhatsApp group (experts). All potential requesters received an information sheet in compliance with UCLA’s Institutional Review Board (IRB) standards detailing the study’s purpose, risks, benefits, and subject rights. They also received instructions about privacy guidelines and expected discussion case formats. The groups required minimal moderation aside from adding and deleting participants and sending reminders to experts if they did not reply promptly.

We obtained approval for this project from UCLA’s IRB (#18-001330). Clearance was also granted by UCLA’s Office of Compliance Services, which deemed it a low-risk project from a professional liability and patient privacy perspective.

## Methods

### Requester surveys

After each case submission, requesting clinicians were expected to fill out a short survey regarding their experience in the discussion groups. Our study team reached out to requesters individually to ensure the survey was completed in a timely fashion. The survey asked for a brief case summary, the level of training of the requester, and three questions about their experience with the case discussion (***Table 2***). The questions were framed as statements with responses on a 5-point Likert scale labeled as (1) Strongly disagree (2) Disagree (3) Neutral (4) Agree (5) Strongly agree.

**Table 2:** Responses to survey questions.


QUESTIONS FOR REQUESTERS	STRONGLY DISAGREE	DISAGREE	NEUTRAL	AGREE	STRONGLY AGREE

My knowledge about a particular medical condition improved as a result of the WhatsApp case discussion.	1	2	7	22	42

	*1.4%*	*2.7%*	*9.5%*	*29.7%*	*56.8%*

I changed elements of my patient’s care based on what I learned from the specialist.	6	6	20	22	20

	*8.1%*	*8.1%*	*27%*	*29.7%*	*27%*

What I learned from the specialist allowed me to implement care which improved my patient’s condition.	13	3	24	11	23

	*17.6%*	*4.1%*	*32.4%*	*14.9%*	*31.1%*

Questions for experts					

The WhatsApp case discussions are exposing me to medical conditions/presentations I rarely see in my current practice environment.	2	5	3	14	24

	*4.2%*	*10.4%*	*6.3%*	*29.2%*	*50%*

The WhatsApp case discussions are augmenting my education about a particular condition or clinical presentation.	2	4	8	25	9

	*4.2%*	*8.3%*	*16.7%*	*52.1%*	*18.8%*


### Expert surveys

Twice during our study period, the expert respondents in each group were surveyed as well. They were asked to respond using the same Likert scale to the statements shown in ***Table 2***.

## Outcomes

From January 2017 to March 2020, our groups discussed 95 clinical cases. Of these, 23 were submitted by members of the study team, and their surveys were excluded from statistical analysis to avoid bias. Sixty-nine of the remaining 72 cases (96%) resulted in completed post-discussion surveys, which were included in the final statistical analysis. The radiology/pulmonology and dermatology groups received heavier traffic than others, accounting for 55% of cases (see ***Table 1***).

Of the 42 individuals who submitted the 69 cases and completed a survey, 34 were UCLA residents and four were UCLA faculty visiting Malawi, usually working closely with residents. Although we hoped Malawian clinicians would participate avidly in the case discussions as well, only four submitted cases. Responses from Malawian clinical and medical officers were comparable and, in some cases, even more positive than those from UCLA residents and faculty.

The requesters gave generally positive feedback, and the strongest trend was towards a positive impact on their learning — well over half of submitters strongly agreed that their medical knowledge improved as a result of the discussion. Remarkably, they also reported an impact on patient care, with more than half reporting they agreed or strongly agreed that the WhatsApp groups changed their management plan (***Table 2***).

The positive trend was present but less strong for patient outcomes. Although a third of respondents strongly agreed that the discussions improved their patients’ outcomes, a similarly sized group said they were neutral, and 18% (12/68) said they strongly disagreed that the discussion had a positive impact on their patient.

Data from the subpopulation of Malawian clinical officers and medical officers is difficult to analyze because there were only four responses, but the trend of their responses is even more positive with regards to educational benefits and patient outcomes. They were slightly less likely to report changing management based on the discussion (see Appendix, Figures 1–3).

The experts also gave positive reviews. The response rate was high among this group as well, with at least one response per group per round of surveys, and 71% of expert participants completed the survey at least once. Half of experts strongly agreed that the cases broadened their clinical exposure, and another 29% chose “agree.” Given that the experts were not our primary learning target, we were pleased to find that 71% of them agreed or strongly agreed that the groups augmented their own education (***Table 2***).

## Discussion

The first aim of our intervention was to augment medical education among clinicians and residents working in Malawi. Our requester survey results indicate that this was successfully accomplished. In addition, although they were not our primary target, we were pleased that the experts also found the experience educational. Subspecialists in high-resource settings often lack exposure to medical conditions that are not common in their region of practice, so this unexpected benefit was welcome.

Second, we were encouraged to find that the WhatsApp groups had a positive impact on medical decision making, with more than half of respondents reporting changes in management in response to case discussions. It is important to note that this result may underestimate the impact of the groups. In many discussions, experts agreed with a requester’s current medical management; often, a requester would reply “neutral” to the survey question if their proposed plan was affirmed without revision. This should not be interpreted as a null result, as the discussion likely increased the requester’s confidence in their original management plan. In a low-resource setting with limited availability of expert guidance, obtaining a confirmatory second opinion on a complicated case is empowering to a clinician and can promote sound clinical decision-making in subsequent, similar cases.

Third, the results suggest only a weak trend toward improved patient outcomes. It is difficult to assess whether the discussions had negligible impact on patient outcomes, or if outcomes are simply hard to assess in this context. Among outpatient cases, lack of consistent follow-up limits clinicians’ ability to observe improvement, especially for visiting residents. Among inpatients, outcomes are often poor, and even expert guidance may not be enough to alter a patient’s course. This is especially true when experts in a resource-rich context have to adjust their idealized treatment plan to suit a requester’s setting.

When comparing the subspecialty groups, dermatology and radiology/pulmonology were the most highly utilized. We considered two possible explanations for the popularity of these groups. First, dermatology and radiology are less commonly included in the curricula of internal medicine residents, our primary requesting group. These trainees may feel more confident with a radiologist’s opinion of a chest film rather than relying on their own interpretation, especially when encountering less familiar pathologies. Second, we suspect that the visual nature of these case submissions, such as X-rays and photographs of skin lesions, were attractive features of the case discussion that made them especially engaging to both the requesters and the experts.

Historically, networks have relied on coordinators to triage requests and forward them to appropriate experts [19]. Our model was more decentralized, with requesters choosing the subspecialty and sending messages directly to that group of experts. This was feasible in part due to the personal relationships between many of the participants on both sides of the network: residents were often communicating from Malawi with the same specialists they interacted with at UCLA. Furthermore, 75% of our experts cited prior clinical global health experience, and some had worked with the Malawian clinicians on previous visits to PIH, which improved their interest, engagement, and retention in the groups. These relationships likely contributed to the quality of our data and the 96% response rate for our requester surveys, greatly exceeding that seen in the literature [7,8]. It is possible that this introduces bias in our data, as survey responses were not anonymized. However, given that over a third of participants were willing to answer negatively to the statement about patient outcomes, it appears their responses were candid.

A limitation of our study is the retrospective, survey-based design. Given the sample size, heterogeneity of cases, and difficulty establishing a control group or pre-existing baseline data, we felt that surveying requesters about their perceptions of outcomes was most practical.

The major challenge in furthering our educational goal is the low rate of participation from Malawian clinical and medical officers. These clinicians only submitted four cases, which is consistent with findings elsewhere in the literature [19]. In our experience, Malawian clinicians at PIH are eager to discuss complicated patients or unusual presentations in person; frequently in the clinic or on the ward, they seek out each other’s input and enjoy discussing patients with visiting residents. Moreover, their survey responses do support the project’s positive impact on their education and on patient outcomes. However, this did not translate to participation in the WhatsApp groups as we expected. Based on informal conversations with the clinicians, we suspect that their participation is hindered by lack of personal familiarity with the UCLA subspecialists and low confidence in their ability to concisely formulate their cases in a forum with a foreign audience. They also face more time constraints than visiting residents, so the process of engaging in case submission and follow-up likely carries a higher opportunity cost. We plan to work more closely with our partner clinicians to improve their confidence in their case summarization skills and to identify ways that the subspecialists can better meet their needs.

We believe that the intimate nature of these groups, augmented by our program’s physical presence in Malawi, enhances these virtual relationships and increases engagement. Other institutions could consider establishing similar discussion groups to support clinicians in resource-limited settings to enhance the medical education of trainees and specialists.

## Additional File

The additional file for this article can be found as follows:

10.5334/aogh.3156.s1 Appendix. Figures 1–3.
