# Supplementary figures and images for: WhatsApp Linking Lilongwe, Malawi to Los Angeles: Impacting Medical Education and Clinical Management
Source: Ann Glob Health. 2021 Feb 18;87(1):20. doi: 10.5334/aogh.3156 (PMC7894367; doi:10.5334/aogh.3156)

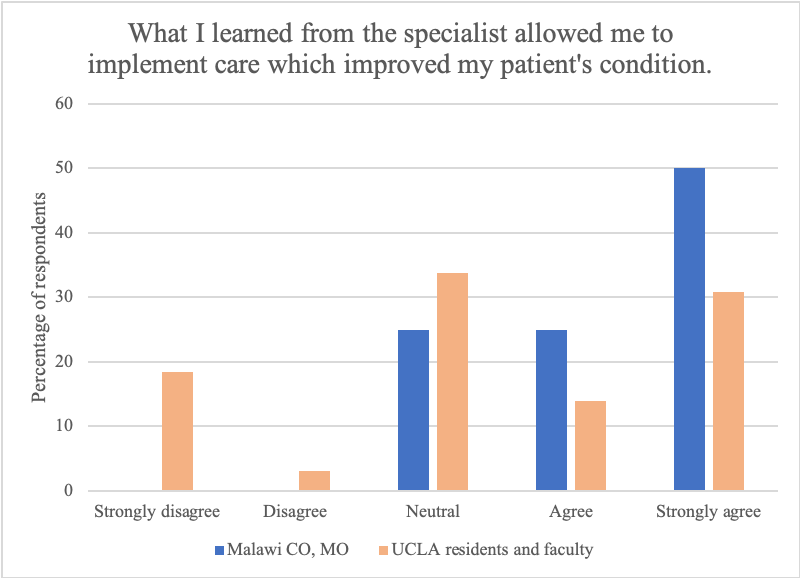

Supplement: Appendix. — Figures 1–3. [file agh-87-1-3156-s1.zip › Figure03.png]

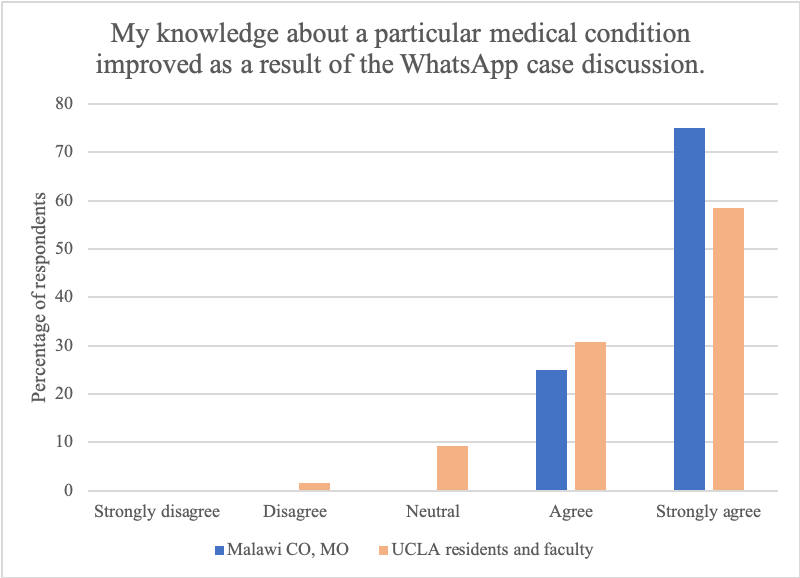

Supplement: Appendix. — Figures 1–3. [file agh-87-1-3156-s1.zip › Figure01.png]

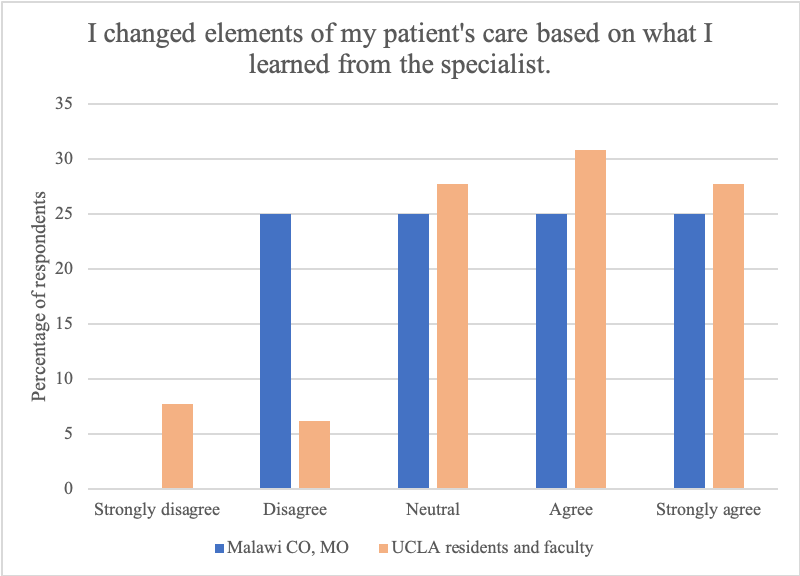

Supplement: Appendix. — Figures 1–3. [file agh-87-1-3156-s1.zip › Figure02.png]
